# *In Vivo* Confocal Microscopy Analysis of the Corneal Layers in Adenoviral Epidemic Keratoconjunctivitis

**DOI:** 10.4274/tjo.59251

**Published:** 2018-12-27

**Authors:** Sevgi Subaşı, Nurşen Yüksel, Müge Toprak, Büşra Yılmaz Tuğan

**Affiliations:** 1 Körfez State Hospital, Ophthalmology Clinic, Kocaeli, Turkey; 2 Kocaeli University Faculty of Medicine, Department of Ophthalmology, Kocaeli, Turkey; 3 Gebze Fatih State Hospital, Ophthalmology Clinic, Kocaeli, Turkey; 4 Ağrı Patnos State Hospital, Ophthalmology Clinic, Ağrı, Turkey

**Keywords:** Adenovirus, epidemic keratoconjunctivitis, epithelial keratitis, confocal microscopy, subepithelial infiltrates

## Abstract

**Objectives:**

To describe the clinical features and microstructural characteristics assessed by *in vivo* confocal microscopy (IVCM) in patients with adenoviral epidemic keratoconjunctivitis (EKC).

**Materials and Methods:**

The study included 20 eyes of 12 patients who presented to the Kocaeli University Medical Faculty, Department of Ophthalmology with complaints of watering, crusting, and stinging, were clinically diagnosed EKC, and were examined by slit-lamp biomicroscopy and IVCM during the prodromal phase and the punctate keratitis, deep epithelial keratitis, and subepithelial infiltration stages of EKC.

**Results:**

While biomicroscopic examination findings were normal during the prodromal period of EKC, IVCM showed an increase in Langerhans cell numbers in the subbasal plexus. After onset of clinical EKC, the punctate epithelial keratitis stage was characterized by findings of hyperreflective cell clusters in the basal epithelium layer, increased accumulation of Langerhans cells in Bowman’s layer, and hyperreflectivity in the anterior stromal layers. In the deep epithelial keratitis stage, the basal epithelial cells displayed peripheral hyperreflectivity and the hyperreflectivity of the anterior stromal surface increased and became more rounded. In the subepithelial keratitis stage, these findings persisted in addition to increased anterior stromal surface hyperreflectivity and focal round plaques.

**Conclusion:**

This study shows that the inflammatory process in the cornea starts in the prodromal period of EKC. Massive inflammation of the epithelium and stroma was observed in the active phase and focal changes were observed on the anterior stromal surface starting in the subepithelial infiltration period.

## Introduction

The most common cause of viral conjunctivitis is adenoviruses. Adenoviral conjunctivitis can manifest clinically as acute follicular conjunctivitis, pharyngoconjunctival fever, epidemic keratoconjunctivitis (EKC), or chronic conjunctivitis. EKC caused by adenovirus serotypes 8, 19, and 37 occurs in epidemics, particularly in the summer months, presents with keratitis in 80% of cases, and shows the most severe clinical course.^1^

EKC is one of the viral diseases that cause severe ocular surface inflammation. After a prodromal period of 7-10 days, unilateral or bilateral follicular conjunctivitis develops; within 2-4 days after onset of conjunctivitis, diffuse epithelial keratitis appears, followed by focal epithelial keratitis. A subepithelial infiltration period begins in the third week, and this clinical presentation may last for weeks or even months.^2,3,4^

*In vivo* confocal microscopy (IVCM) is a non-contact imaging method that enables evaluation of the cornea at the cellular level.^5^ In addition to having a well established place in the diagnosis and follow-up of many corneal diseases, studies including IVCM findings have also shown corneal changes in the various stages of EKC. These studies described changes starting at the basal epithelium level and extending into the midstroma, while images targeting the subepithelial infiltration period showed focal inflammatory foci.^6,7^ In this study we sought to use IVCM to elucidate corneal alterations that begin in the prodromal period of EKC, evaluate findings seen in the clinical course of the disease, and discuss our results within the context of the literature.

## Materials and Methods

The study included 20 eyes of 12 patients (6 males, 6 females) who presented with complaints of burning, watering, and discharge from the eyes and were clinically diagnosed with EKC in the ophthalmology outpatient clinic of the Kocaeli University School of Medicine. Ethical approval was obtained from the university ethics committee, and informed consent was obtained from all participants prior to examination.

Following clinical assessment with biomicroscopy, patients underwent IVCM (Rostock Cornea Module/Heidelberg Retina Tomography 3, Heidelberg Engineering GmBH, Germany) examination under topical anesthesia (0.5% proparacaine Hydrochloride; Alcaine^®^; Alcon Laboratories, Fort Worth, TX, USA). A new sterile polymethylmethacrylate cap (Tomocap^®^; Heidelberg Engineering GmBH, Germany) was placed over the objective lens for each patient. Gel (Viscotears^®^; Carbomer 980, 0.2%; Novartis, North Ryde, Australia) was applied to the cap at the start of imaging. The distance between the cornea and objective was monitored on the camera display as imaging was initiated. After visualizing the surface epithelium on the screen, the objective lens was manually focused to acquire images of the corneal layers sequentially until reaching the endothelium.^8^

At initial examination, patients underwent IVCM both in the eye diagnosed with EKC and the eye with no clinical signs. IVCM imaging was done in the patients’ healthy, non-EKC eyes at each follow-up visit in order to capture images in the prodromal period. For the patients whose healthy eyes developed clinical EKC during follow-up, eyes imaged by IVCM within the 7-10 days prior to the appearance of EKC signs were evaluated as prodromal (4 eyes), while eyes that did not develop clinical EKC and remained healthy throughout follow-up were evaluated as the control group (4 eyes). Of the imaged eyes with clinical disease, the routine ophthalmologic examination findings, anterior segment photographs, and IVCM findings of 4 eyes with punctate epithelial keratitis, 4 eyes with deep corneal keratitis, and 4 eyes with subepithelial infiltration were evaluated. Slit-lamp microscopy findings and disease stages were recorded. IVCM findings were scored as 0 (same as control), + (slight increase compared to control), ++ (moderate increase compared to control), and +++ (extreme increase compared to control).^6^ All assessments were done at different stages in different patients; disease stages in which patients were examined are shown in Table 1. Patients with history of any ocular disease or with any chronic systemic disease were not included in the study. All eyes with active clinical EKC were treated with topical 0.3% tobramycin (Tobrased, Bilim İlaç, İstanbul, Turkey) 6 times a day and preservative-free artificial tears (Tears Naturale Free, Alcon) 8 times a day. None of the patients in the study were treated with steroids. All treatment except preservative-free tears was discontinued when clinical symptoms had resolved, after about 14 days of treatment.

## Results

**Figure 1:**
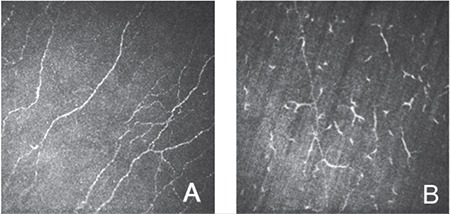
Appearance of subbasal plexus in healthy cornea (A) and increased Langerhans cells in the subbasal plexus in the prodromal period (B)

Clinical features, disease stages, slit-lamp examination findings, and IVCM findings of the patients are given in Table 1. In eyes examined in the prodromal period before the onset of clinical EKC, the epithelial, Bowman’s, and stromal layers appeared normal in IVCM, while the subbasal plexus showed an increased number of Langerhans cells (Figure 1).

**Figure 2:**
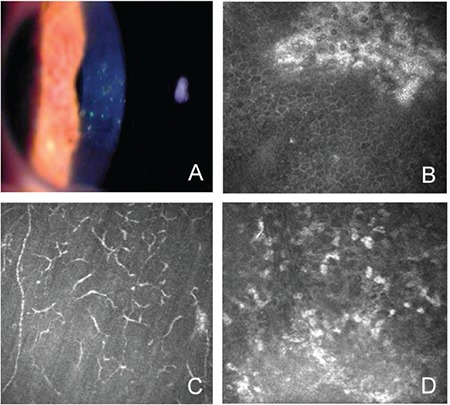
Fluorescein-stained foci of punctate keratitis in the cornea (A); cell clusters in the basal epithelial layer (B); increased Langerhans cells (C); and hyperreflectivity in the anterior stroma (D)

Clinical EKC eyes evaluated during the punctate epithelial keratitis stage showed cell clusters surrounded by inflammatory cell infiltration in the basal epithelium. An increased number of branching dendritic cells were observed in Bowman’s layer. Hyperreflective cells were noted in the anterior stroma (Figure 2).

**Figure 3:**
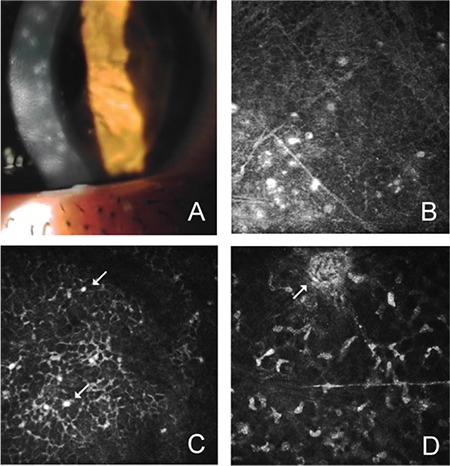
Foci of deep epithelial keratitis (A); hyperreflective inflammatory cells between the basal epithelium and anterior stromal surface (B); basal epithelial cells and inflammatory cells (arrows) with peripheral hyperreflectivity (C); and inflammatory focus in the anterior stroma (arrow) (D)

Eyes in the deep epithelial keratitis stage showed basal epithelial cells with peripheral hyperreflectivity in keratitis foci, inflammatory cells in the form of punctate hyperreflectivity, and the hyperreflective areas in the anterior stroma had acquired round focal borders. The increase in Langerhans cells in the subbasal plexus continued (Figure 3).

**Figure 4:**
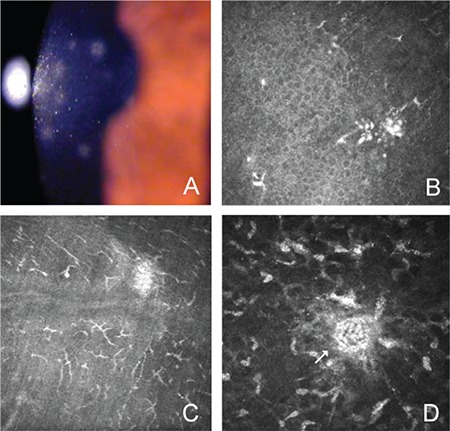
Areas of subepithelial infiltration (A); focus of hyperreflective keratitis in the basal epithelium (B); Langerhans cell connections in the subbasal plexus (C); and focal hyperreflective plaque with round border in the anterior stroma (arrow) (D)

In the subepithelial infiltrate period, the basal epithelium still exhibited hyperreflective foci and inflammatory cells, but the areas of anterior stromal hyperreflectivity formed more distinct round hyperreflective plaques. The eyes exhibited no changes in the deep stromal layers or endothelium during the course of EKC (Figure 4).

## Discussion

The cornea is the most densely innervated tissue in the body, and this innervation provides corneal sensitivity. Many diseases disrupt corneal sensitivity, including ocular infections, herpetic eye disease, dry eye syndrome, and diabetes.^9,10,11,12,13,14^ Animal studies have shown a correlation between corneal inflammation and innervation.^15,16^ Hamrah et al.^17^ and Liu et al.^18^ demonstrated that immature dendritic cells in the cornea had matured after inflammation and transplantations. In noninflammatory, quiet conditions, dendritic cells are found in the central corneal epithelium and anterior stroma, whereas during inflammation they infiltrate the entire cornea, thus preparing it to respond to pathogens.^19^ With IVCM enabling *in vivo* visualization of these cells, it has become possible to document their increase in immune active situations.

Corneal involvement occurs during the course of EKC, and various corneal findings can be observed in the different disease stages. Corneal involvement leads to symptoms such as dry eye, glare, blurry or low vision, and irregular astigmatism.^20^ No corneal and conjunctival findings occur in the prodromal period, but clinical signs of conjunctivitis appear within 7-10 days after this period. Despite apparently normal biomicroscopic and clinical findings during the prodromal period, IVCM revealed a marked increase in Langerhans cells in the subbasal plexus in our study, indicating that inflammation has already started. These findings suggest an active prodromal process in the healthy eye that precedes clinical disease.

The active follicular conjunctivitis phase is characterized by the formation of corneal punctate epithelial keratitis, followed by a long-term inflammatory process with subepithelial infiltration, believed to be a result of type 4 hypersensitivity reaction. In EKC, inflammatory cell infiltration in the basal epithelium and anterior stromal surfaces has been demonstrated by the higher concentration of dendritic cells observed in IVCM.^6,7,21^

The increase in dendritic cells in the subbasal plexus is considered an important IVCM finding in EKC and herpes simplex keratitis. Öztürk et al.^22^ reported that herpetic keratoconjunctivitis can often be confused with adenoviral EKC due to similarities in their clinical course and common IVCM findings. In addition, a temporary reduction in corneal sensitivity has been observed following inflammatory cell activation in 74% of patients with EKC.^22^

In the subepithelial infiltration phase, an increase in inflammatory cells is observed in addition to inflammatory foci in the stroma. Dosso and Rungger-Brandle^6^ reported that Langerhans cells were reduced in more advanced disease stages. However, our study encompassed the earlier subepithelial infiltrate stage and showed an increase in Langerhans cells, consistent with the literature.

## Conclusion

Our study demonstrates based on IVCM findings that corneal involvement in EKC begins not in the clinical disease stage but in the prodromal phase, with an increase of Langerhans cells. In clinical disease stages, findings such as increased dendritic cells accompanying the development of epithelial keratitis, and hyperreflective plaques in the basal epithelial layer and anterior stromal surface are seen on IVCM. In the subepithelial infiltration phase, lesions become more focal and persist without extension to the posterior stromal surface. Based on our findings, we suggest that corneal findings in IVCM signal the development of clinical EKC starting in the prodromal period.

## Figures and Tables

**Table 1:**
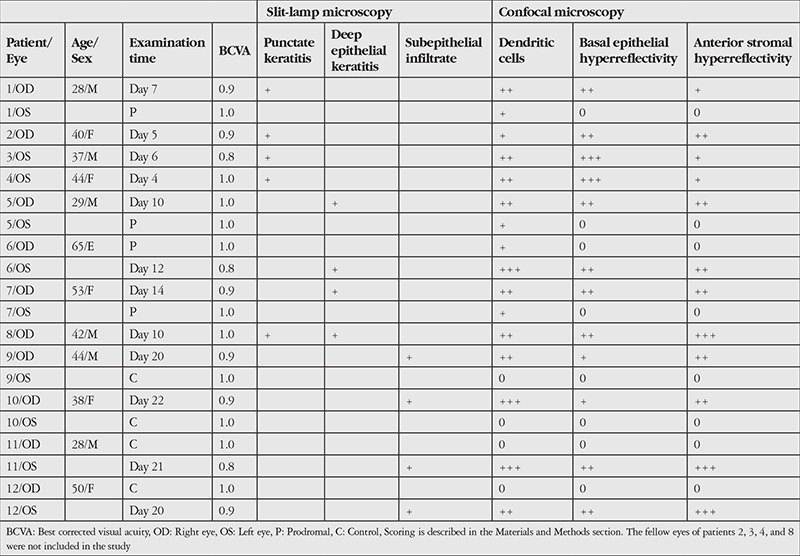
The patients’ clinical, slit-lamp microscopy, and *in vivo* confocal microscopy evaluations
